# Social media and digital technology use among Indigenous young people in Australia: a literature review

**DOI:** 10.1186/s12939-016-0366-0

**Published:** 2016-05-25

**Authors:** Emma S. Rice, Emma Haynes, Paul Royce, Sandra C. Thompson

**Affiliations:** Georgetown University, 3700, O St NW, Washington, DC 20057 USA; Western Australian Centre for Rural Health (WACRH), University of Western Australia, 167 Fitzgerald St, Geraldton, WA 6530 Australia

**Keywords:** Social media, Digital technology, Indigenous, Aboriginal, Youth, Young people, Australia

## Abstract

**Introduction:**

The use of social media and digital technologies has grown rapidly in Australia and around the world, including among Indigenous young people who face social disadvantage. Given the potential to use social media for communication, providing information and as part of creating and responding to social change, this paper explores published literature to understand how Indigenous Australian youth use digital technologies and social media, and its positive and negative impacts.

**Methods:**

Online literature searches were conducted in three databases: PubMed, Google Scholar and Informit in August 2014; with further searches of additional relevant databases (Engineering Village; Communication & mass media complete; Computers & applied sciences complete; Web of Science) undertaken in May 2015. In addition, relevant literature was gathered using citation snowballing so that additional peer-reviewed and grey literature was included. Articles were deemed relevant if they discussed social media and/or digital technologies and Indigenous Australians. After reading and reviewing all relevant articles, a thematic analysis was used to identify overall themes and identify specific examples.

**Results:**

A total of 22 papers were included in the review. Several major themes were identified about how and why Indigenous young people use social media: identity, power and control, cultural compatibility and community and family connections. Examples of marketing for health and health promotion approaches that utilize social media and digital technologies were identified. Negative uses of social media such as cyber bullying, cyber racism and the exchange of sexually explicit content between minors are common with limited approaches to dealing with this at the community level.

**Discussion:**

Strong cultural identity and community and family connections, which can be enhanced through social media, are linked to improved educational and health outcomes. The confidence that Indigenous young people demonstrate when approaching the use of social media invites its further use, including in arenas where this group may not usually participate, such as in research.

**Conclusions:**

Future research could examine ways to minimise the misuse of social media while maximising its positive potential in the lives of Indigenous young people. Future research should also focus on the positive application of social media and showing evidence in health promotion interventions in order to reduce health inequities between Indigenous and non-Indigenous young people.

## Introduction

The world is changing rapidly as a result of new digital technologies which have revolutionised opportunities for and the nature of networking and communication. It is essential to understand how different populations use digital technologies and social media in order to explore their potential to improve social and health outcomes.

Social media refers to social interaction among people in which they create and share information and ideas in virtual communities and networks. Utilising digital media and web-based platforms enables “users to actively share information, generate content, collaborate and interact with each other…[and] can be accessed from computer and mobile technologies” [1]. An important component of social media is the role of the audience as a user who engages actively with the content rather than simply viewing it [2]. The term social media covers a range of different platforms with overlaps between the different types which include: Social Networking such as Facebook and Linked In (which enable connections with other people of similar interests and backgrounds and allow creation of a profile, various ways to interact with other users and ability to setup groups); Media Sharing (such as YouTube and Flickr that enable uploading and sharing of media such as pictures and video); Microblogging (such as Twitter which provides short updates pushed out to anyone subscribed to receive the updates); and Blogs (which are online forums where members hold conversations by posting messages, with discussion around the topic of the blog post). This paper includes a discussion of social media and digital technologies that are used to access social media and uses the terminology “social media” when discussing the topic broadly.

Digital technologies, such as mobile smartphones and laptop computers, allow users to access social media easily and frequently and have substantially increased the uptake of social media. Social media is a relatively recent phenomenon as ownership and access to computers, smart phones, tablets and the Internet has rapidly increased in Australia. Facebook reported 13.6 million Australian users in September 2014 compared to only 12 million in September 2013 [3, 4]. Adolescents and young adults are more likely to use social media than older sectors of the population [2], with social media usage increasing from the younger to the older teenage years [5] and Facebook usage among the Aboriginal and Torres Strait Islander (hereafter termed Indigenous) population higher than in the Australian population overall (Table 1) [6] and personal communication Matt Balogh). These statistics demonstrate a changing world in which communication and engagement with the world via social media channels has become common, particularly in young people and including in Indigenous Australians. Youth or young people refers to people in that period between childhood and maturity, often called youth and is considered to be those between puberty and 25 years; however, it is often used as a term without being explicitly defined. The Indigenous population distribution is much younger than the Australian population generally, with a median age of 20 years compared with 35 years, and Indigenous Australians are more likely than other Australians to live in small, remote communities, with relatively few services, facilities, and opportunities [7]. Those people that live more remotely were generally colonised later and show less acculturation to western ways and greater adherence to cultural traditions and speaking native languages other than English.

**Table 1:** Social media use in Australia

Population group	Year	Type of usage	% of population that are users	Source of Data
12–13 year old Australians	2013	Use of a social networking site in the last four weeks	67 %	ACMA Research Report [43]
14–15 year old Australians	2013	Use of a social networking site in the last four weeks	85 %	ACMA Research Report
16–17 year old Australians	2013	Use of a social networking site in the last four weeks	92 %	ACMA Research Report
All Australians	2014	Facebook	42 %	McNair Ingenuity Research Institute reported in [6]
Aboriginal and Torres Strait Islander Australians	2014	Facebook	60 %	McNair Ingenuity Research Institute

Many Indigenous young people in Australia face social disadvantage, particularly in the areas of education, child safety and criminal justice system involvement. It is common for Indigenous young people to drop out of school, with only 25 % of Indigenous people aged 15 years and over reporting their highest educational level achieved as Year 12 or equivalent compared to 52 % of the non-Indigenous population [8]. Disparities are evident from the first year of schooling and remain apparent throughout the schooling years. Contributing to the less developed literacy and numeracy skills of Indigenous students is their lower attendance at school so working collaboratively with Indigenous communities to develop practical and relevant strategies is necessary to improve the educational status of Indigenous people [9]. Indigenous young people are more likely to experience abuse, with the rate of assault among Indigenous children around 5 times higher than among non-Indigenous children [10] and rates of juvenile incarceration are 31 times that of non-Indigenous youth [11]. This combination of educational disengagement, child safety issues and criminal justice system involvement are part of the complex web of social disadvantages that many Indigenous young people face and important social determinants of health inequities, “the unfair and avoidable differences in health status seen within and between countries” [12]. Addressing social disadvantage is therefore essential to reduce health inequities in the Australian Indigenous population.

Many Indigenous Australians have utilised the Internet from its early days [13, 14], despite the economic, social, cultural and geographic factors that can affect their access. With increasing access and use of social media, understanding its use and impacts among Indigenous young people could contribute to both interventions and to monitoring and evaluation of programs. Media and social marketing have long been part of health promotion related interventions and there is evidence that such interventions can work with a range of target groups, in different settings, and can work upstream as well as with individuals [15]. In the same way, social media is now commonly proposed as part of interventions. However, it is essential to understand how a target group uses and responds to a technology in order to use if effectively, and to ensure that any proposed use is appropriate and does not lead to adverse effects. This paper explores published literature on the ways in which social media is used by and for young Indigenous Australians, acknowledging their diversity, and with a view to positive and negative impacts and how this potentially impacts health and health promotion approaches.

## Methods

**Fig. 1:**
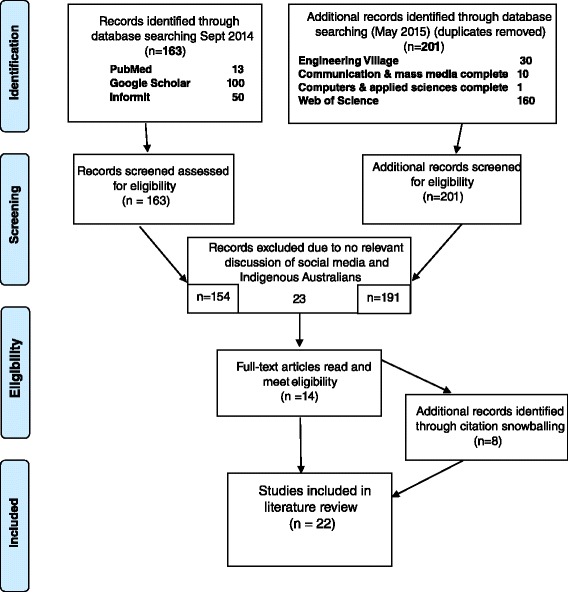
Search methods and processes used in the literature review

Initially, online literature searches were conducted in the following three databases: PubMed, Google Scholar and Informit. Informit provides access to specialist content through over 80 databases with subject-based databases featuring coverage of Australian and international information resources covering a wide range of subjects, including health, engineering, business, education, law, humanities and social sciences. During August 2014, searches of these three databases were progressively undertaken using the following search terms: (indigenous [mesh] OR oceanic Ancestry Group [mesh] OR aborigin* OR indigeno* OR indigene*) AND (social media [mesh] OR Facebook OR social networking OR virtual communications OR online networks OR cyberbull* OR DivaChat OR Twitter OR YouTube) (Fig. 1). The initial search was screened for relevance by reading the titles and abstracts with the full article then obtained for further full-text assessment. A number of papers that referred to use of internet and social media only related to Indigenous student learning in higher education were excluded. Citation snowballing was used to identify additional relevant sources. Grey literature, which refers to academic literature that is not formally published such as monographs and technical reports from government agencies or scientific research groups, working papers from research groups or committees, was also included. Articles were deemed relevant if they discussed social media and/or digital technologies and the Australian Indigenous population. Only articles that referred to humans and Indigenous populations from Australia were included.

The PubMed search for articles published in the last 10 years produced 2 relevant articles with a further four sources identified through citation snowballing. The Google Scholar search for articles since 2010 produced 4,620 results sorted by relevance, of which three relevant articles were identified in the first 50 of the 100 results reviewed, with two additional sources identified through citation snowballing. The Informit search produced 940 results sorted by relevance, of which four articles were included from the first 50 results identified and two additional sources included as a result of citation snowballing. In May 2015 a further search of additional databases (Engineering Village (journal articles only); Communication & mass media complete; Computers & applied sciences complete; Web of Science) resulted in identification of 201 potential references which after screening and elimination of already included duplicates resulted in an additional 6 articles, two published since the previous 2014 search. Several articles were eliminated based upon their content being restricted to on-line learning of Indigenous tertiary students.

After reading and complete review of all of the relevant articles, the issues explored by the papers were refined and grouped, forming an emergent thematic framework which was used to identify the overall themes; specific examples were identified. The papers/sources were reviewed by at least two reviewers to ensure agreement upon the main themes identified in this paper.

### Findings

The familiarity that many Indigenous young people have over modern technology is reported as giving them a sense of fearlessness and control when approaching the use of new platforms [1, 16–18]. Uptake and access to mobile devices and the Internet has led to widespread use of social media among many Indigenous youth, providing an opportunity for them to participate and communicate in new ways. The availability of satellite rather than the previous and rudimentary dial-up connections has allowed Internet and mobile phone services to reach even remote areas in Australia. Although the living situations and other factors of Indigenous youth differ widely across Australia, even those who lack food and clothing may still have their own Smartphone [19]. The widespread use of and familiarity with social media even among socially disadvantaged Indigenous young people and the priority young people place on a Smartphone highlights its importance, although there is little information on how usage differs by age and geographic location [19].

The 22 publications identified came from both academic (*n* = 14) and grey (*n* = 8) literature, as non-peer reviewed material was drawn upon in the academic research on social media usage among young Indigenous Australians. Several themes were identified within the literature: identity; power and control; cultural compatibility; and community and family connections. Although these have been identified as separate themes, it is important to note that many of them overlap and are interrelated. A summary for each article is provided in Table 2 with information according to the type of source: academic literature (journals, reports, academic articles) or grey literature (website documents). Within these two categories, the sources are listed in the chronological order. In general, academic sources discussed social media in terms of the identified themes and/or policy discussions, while the grey literature was more likely to refer to specific programs using social media.

**Table 2:** Literature review article summary: peer-reviewed and grey literature

Peer-reviewed Literature (including articles identified through database searching and citation snowballing)					
Author/Year	Study Population/Topic Details	Type of Media	Methods/Limitations	Positive Impacts of Social Media	Negative Impacts of Social Media
Kral I (2010) [16]	Indigenous youth participating in non-formal community-based media projects in remote communities	Radio, television	Ethnographic case study data	-availability of satellite rather than dial-up connections has allowed the Internet and mobile phones to reach remote areas -access to internet and media available at communal spaces such as youth programs or media centres -communication via social networking or SMS text messaging using text and symbols in inventive ways that reflect local culture and dialects of speech -sense of familiarity and control over modern technology -increased sense of belongingness to globalized youth culture	Not discussed
Lumby B (2010) [17]	26 current Indigenous university students or graduates who maintain Facebook profiles	Facebook	Methods: Interviews	-allows Indigenous people to identify and share their Indigenous identity online -“Facebook acts as a modern site for kinship and connectivity”	-pressure to prove indigeneity and point out those who are ‘faking’ it due to the surveillance aspect of Facebook -“Facebook…acts as a restraining force that regulates who can and who cannot ‘be’ Indigenous”
			Limitations: -limited due to small sample size -does not report the study in full		
Kral I (2011) [18]	Indigenous youth uptake of new media technologies and implications for cultural practice	“new media technologies”	Ethnographic case study data	- youth increasingly in control of production and distribution of media content - youth increasingly comfortable to have their images/names in public space (less “shamed”) - new kinds of agency in learning enabled - increased social mobility due to changed agency/disposition and opportunities available through the internet -increased capacity to collaborate with researchers, increased reflexivity as a result	Not discussed
Petray T (2011) [23]	Focus on the use of social media as tool for protest and activism amongst Aboriginal people in Townsville	Listserv, Facebook,	Does not stray too far from the key theme that social media is an effective tool for activism… focus is on the general use of social media by older Aboriginal people	Digital democracy - de-centres the role of the expert and places emphasis on the conversation –people can exercise their right to communicate, and in their own language, to an outside audience.	Not discussed
				Online networks enable people to create and present their identity to others.	
				Share information across a wide audience to motivate others to participate in action	
Healy JDL (2013) [13]	Young Aboriginal men from Arnhem Land and wide discussion of Aboriginal media in general	YouTube, video, radio, television, Internet, Facebook, Diva Chat	Anthropological	-early involvement of Indigenous people in the Internet, partially due to multimedia visual and oral nature -ability to use Internet without control from non-Indigenous people -content-sharing platforms online provide opportunities for Indigenous people to transmit intergenerational knowledge and explore and express their modern selves	Not discussed
	Time Period: Video clip filmed in September 2007				
Sweet M, et al. (2013) [24]	@IndigenousX Twitter account	Twitter	Case study	-provides an opportunity for Indigenous people to participate in the public forum, share stories, and challenge stereotypes	Not discussed
Sweet MA (2013) [28]	Not discussed	Not discussed	Social media based health promotion campaigns	-cited examples of successful programs that use social media, including the No Smokes Campaign, @IndigenousX Twitter account, the Rewrite Your Story initiative, HITnet, Young and Well Cooperative Research Centre virtual mental health resources	Not discussed
Rae K, et al. (2013) [32]	Participants in the ArtsHealth program focused on pregnant mothers	Facebook	Complex recruitment methods with heavy involvement from local community	-participants were more responsive in communications via a Facebook inbox compared to a phone call or text message -integrating social media into the research approach was very successful for the “rural” setting	-Internet access becomes increasingly difficult as remoteness increases
	Time period: Implemented in 2009				
Herborn D (2013) [34]	Cyber racism and policy	Twitter, Facebook, newspaper article user comments	Policy discussion	Not discussed	-cyber racism as a negative impact of social media -policy is difficult to enforce → the Australian government has done little to try and take down user-generated content that is in violation of the Racial Discrimination Act
Petray, T (2013) [25]	Social media and activism by Aboriginal people in Australia	Twitter	Social activism using social media	-Social media is inexpensive, immediate and broad reaching -Promotes individual and collective identities - collective identity promotes collective agency -Social media can counter essentialised understandings of Aboriginal people initiate collective action against a perceived inequality -Social media contests stereotypical identifications, creates identity and is self-forming enabling anyone to write to a broad audience	Not discussed
Brusse K et al. (2014) [26]	Review article examining peer-reviewed evidence of benefit for social media and mobile technologies used in health promotion, intervention, self-management, and health service delivery	Social media, smart phones and text messaging	Systematic review of peer-reviewed literature, focused on health promotion	Noted the unique capacity of social media to reach Indigenous Australians as well as other underserved populations because of their wide and instant disseminability	Evidence of social media strategies being effective in health promotion for Indigenous populations is limited.
Kral I (2013) [44]	Indigenous youth uptake of new media technologies and changes in social interaction and communication	“new media technologies”	Ethnographic case study data	- technologically assisted communication becoming normalized, even when literacy is low - changes in social interaction, communication and ways of being – increasingly comfortable in a more ‘public’ online world - youth create as well as consume – appropriating technology for own sociocultural purposes	Technology has disturbed traditional communication – increasingly only peer-to-peer, less intergenerational communication
Radoll P (2014) [14]	Not discussed	All ICTs	Not discussed	-use of Smartphones and mobile devices even in communities where there is no mobile phone service → people use it as a multi-media device and then connect to the internet when it’s available	-potential for sex-texting and cyber bullying, which is hard to monitor → best way to address these issues is through educational programs such as *Be Deadly Online*
Vaarzon-Morel P (2014) [20]	Lander Warlpiri settlement from Willowra	Radio-television, video, television, radio, mobile phones, other portable digital devices, Diva Chat	Anthropological	-use of mobile phones to “intensify family intimacy” and stay connected to kin who may live far away -continuities between technology and Warlpiri culture, such as mobile phones being viewed as extensions of person and often shared by family members	- difficult for elders to regulate relationships along skin groups as per traditional way -increased connectivity means that conflicts that previously remained local have the ability to spread through inform kin in other places about conflicts -use of Diva Chat for illegal sex-texting among teenagers, cyber bullying, and posts that are meant to incite violence between feuding families
	Time Period: 1970s-present				
Grey Literature					
Author/Year	Study Population/Topic Details	Type of Media	Methods/Limitations	Positive Impacts of Social Media	Negative Impacts of Social Media
Edmonds F, et al. (2012) [1]	Young Aboriginal people in Victoria	Mobile phones, technology, and social media	-Lit review -formed an Aboriginal reference group -small group discussion -thematic analysis of transcripts	-Mobile technologies allow opportunities for alternative forms of learning and literacy, such as digital storytelling -mobile phones and social media used to stay connected to people -discussion of identity and media literacy	-e-learning in schools needs to be culturally sensitive to Indigenous students who may not have computers or internet at home and therefore may be less familiar with technology than other students
	Time period: March 2012				
	Age: 12–24 years				
	Gender: 8 males, 3 females				
			Limitations: -Very small sample size		
No Smokes (2012) [27]	No Smokes Campaign	Interactive website linked with social media platforms such as Facebook and YouTube	Health promotion campaign	-developed using input from Indigenous young people → youth more drawn to multimedia, video, social networking, animation, music and mobile phones than traditional anti-smoking marketing campaigns → more widely accessible media forms because they help minimize language and literacy barriers	Not discussed
	Target population: Indigenous young people				
Joint Select Committee on Cyber-Safety (2013) [19]	Cyber safety for Indigenous Australians	Internet, mobile phones, Facebook	Report	Not discussed	-factors such as socioeconomic status, family structure, education level, and employment status can serve as barriers to ICT use -generational gap in knowledge and use of mobile technology and social media between Indigenous youth and their parents and elders so cyber bullying can go on unaddressed → need for educational programs
Hitnet (2014) [29]	Interactive kiosks in remote areas to address issues such as smoking, STIs, teenage pregnancy, depression, and suicide	-kiosks that use audio-visual touch interfaces - kiosks include connections to Facebook, Twitter, and YouTube that can update local community content and applications that can be down-loaded to personal mobile devices	Health promotion	-utilizes multimedia nature of technology and social media -ability to connect with remote areas -ability to distribute and download content and incorporate local content uploaded by users	Not discussed
Rewrite Your Story (2014) [30]	Anti-smoking initiative focusing on 16 local community ambassadors who share their stories via video about how and why they quit smoking	Online videos, Facebook, Twitter	Health promotion campaign	-utilizes the idea of rewriting your story by communicating and sharing via other social media channels such as Twitter and Facebook	Not discussed
Cairnduff S (2011) [31]	It’s Your Choice! Have A Voice! campaign	Facebook	Health promotion campaign	-active engagement, information sharing and activity on Facebook page to improve choices about sexual and reproductive health	Not discussed
Cherbourg Mojo Project (2013) [33]	Indigenous Youth job seekers with low levels of literacy and numeracy	iPhones (digital storytelling)	10-week mobile journalism training pilot program	-incorporating media into education through mobile journalism and digital storytelling -youth participants gained job skills and improved their reading, writing, and math abilities	Not discussed
	Time Period: May-July 22013				
	Age: 15–24 years				
Kral (2013) [44]	Indigenous youth uptake of new media technologies and implications for cultural practice	“new media technologies”	Ethnographic case study data	- History of use of technology - Extended social connections - Increased reflexivity - Learning beyond school	

### Identity

The use of content-sharing platforms and user-generated content online, such as on Facebook and YouTube, provides opportunities for Indigenous young people to ‘perform’ their Indigenous identities online [1, 13, 16, 17, 20, 21]. Sharing stories or videos online or being part of Indigenous Facebook groups provide ways for Indigenous young people to connect with, affirm and give voice to their Indigenous identities. Social media gives Indigenous young people the opportunity to present their Indigenous identity to others, which helps them further define and affirm that identity [13, 16, 17]. Facebook in particular can serve as a way to confirm and enact Indigenous identity through use of particular language, iconography or images, membership in certain groups or organizations and by being Facebook ‘friends’ with other Indigenous people [17]. On the other hand, it has resulted in a pressure to ‘prove’ one’s indigeneity, especially for individuals who do not ‘look’ Indigenous, which Lumby argues is an example of how Facebook can act “as a restraining force that regulates who can and who cannot ‘be’ Indigenous, and indeed what it means to be Indigenous” [17]. Ginsberg highlights the way in which control may be asserted in film-making to ensure that real rather than fictional stories are told so that film tells real stories using real voices [22]. Kral, reporting on long term ethnographic research, identifies a transformation in Indigenous youth who are less ‘shamed’ then previous generations and more comfortable with having their names or images in the public sphere [18].

### Power and control

Social media provides opportunities for Indigenous young people to feel a sense of power and control over their own identities and communities [18] . They can do this even in some communities where there is no mobile phone service through using Smartphones and mobile devices as multi-media devices and then connecting to the Internet when in areas where it is available [14]. Mobile phones with cameras and web browsers have expanded opportunities for Indigenous people to create media recordings and to share information via social networking sites such as Facebook or AirG Diva Chat [13, 20].

One reason why many Indigenous young people have embraced social media is its self-directed nature where users can produce their own unregulated content. Indigenous young people can participate and use social media without any control or input from adults or from the non-Indigenous community that controls the larger, more conventional media forms [13, 14, 17]. This self-directed nature also means that Indigenous young people can seek out information for themselves, enabling new forms of agency [18, 21] . Some health programs aim to harness this self-driven ability to access resources, as is discussed in the section on social marketing and health promotion. Indigenous activists have extended their efforts beyond listserves and blogs to utilise social networking sites to make their struggle known to a wide audience as part of their social movement [23].

Earlier media forms such as production equipment often required special resources, permission and oversight for young people to use. However, with the advent of affordable digital technologies such as mobile phones and laptops, the power of media is now in the hands of people themselves. The control that this gives Indigenous young people over social media provides the opportunity for them to represent themselves rather than having “the other” record and represent them [1, 13, 16, 17, 22]. The nature of media itself allows people with low literacy to grasp an intuitive understanding of the technology because many digital processes can be remembered spatially and incorporate a creative icon-based approach which layers images, sounds, texts and symbols. This is especially important in Indigenous populations where young people may be below the national standards for literacy [13, 16].

### Cultural compatibility of multimedia

The involvement of many Indigenous young people in social media is partially due to the multimedia nature of the medium which lends itself to the orally and visually focused cultures of Indigenous communities rather than Western-based literacy and numerics [13, 16]. Social media also has the potential to support Indigenous priorities of community and communication, with some Indigenous leaders identifying the mesh of interactions present in social networks as similar to ancient imagery and ancient communication channels [20, 24]. The sharing of information online related to Indigenous identity and culture also provides opportunities for transmitting intergenerational knowledge within and between Indigenous communities, an important aspect of Indigenous culture. For example, a film that features Elders discussing Indigenous stories, traditions, etc. can be shown to younger Indigenous people and potentially to future generations; this is a modern, social media-based form of oral tradition. Social media is seen to “allow for the continuation, expansion, and transformation of various ‘traditions’, from traditional language to traditional activism” [25].

Indigenous young people have also been reported communicating via social networking or SMS text messaging using text and symbols in inventive ways that reflect their local culture and dialects of speech [1, 16]. For example, short cut messaging lends itself well to ‘Roper River Kriol,’ an English-based creole found in the Katherine region [16]. Similarly, as noted by Petray, Indigenous activists can embrace the ambiguity offered by social media to challenge mainstream imagery and self-write alternative understandings about what it means to be Indigenous [25].

### Community and family connections

Social media can help form communities that people may not otherwise have the opportunity to connect with. Alternatively, community can be exemplified and strengthened through social media for groups that are already connected. This two-way relationship between social media and community is especially evident among Indigenous young people. Groups on social media can be collections of individuals to form a community, such as a Facebook Group, or group accounts that portray an Indigenous identity [1, 17].

One example of a group approach that aims to provide a media space for Indigenous people to share their stories is the @IndigenousX Twitter account, which is a rotating account with a different Indigenous person tweeting every week. Sharing through public social media forums such as the @IndigenousX Twitter account can provide opportunities for Indigenous people to participate in the public forum. The account was nominated for a Shorty Award, which recognizes achievement in social media. The main reasons identified for the nomination were “that the account shares Indigenous knowledge and stories, challenges stereotypes and reflects the diversity of Indigenous peoples” [24]. This suggests that while the individual users of the @IndigenousX Twitter account may not know each other in real life, they form an online community through their group participation on social media.

Social media also helps connect distant family and friends, even those who have never met in person or who lost touch at some point in the past. Given the mobility of Indigenous people, Facebook in particular serves as a platform for Indigenous young people who have moved to reconnect with their Indigenous identities through groups and pages where people can share their ideas, thoughts, events, music and photos and through connecting with friends and family. According to Lumby, “Facebook acts as a modern site for kinship connectivity and continuity” and as noted by many researchers, digital technologies may act to strengthen kinship connectivity [13, 17, 20]. Many Indigenous young people use Facebook to keep up with family and friends. This sense of support, connection, and community may help “reinforce young people’s mental health and wellbeing” [17].

Real life communities can work together and strengthen their bonds through social media. Social networking sites are being increasingly used among Indigenous young people for communication and for uploading content, rather than more traditional text-based interactions such as emails [13, 14, 16]. In remote areas, this production of user-generated content among Indigenous young people is largely due to the strong presence of youth-oriented programs in non-school hours that provide spaces and opportunities to use media (termed communal ‘digital bedrooms’) and to the widespread availability of mobile phones [16]. Access to computers and the Internet may not be available in homes in remote communities, but they may be available at communal spaces such as programs for young people or media centres [16]. However, some Indigenous families do not have computers or Internet access in the home so mobile phones are the preferred online platform for most Indigenous people to access the Internet and use social media [19].

Although the significance and cultural adaptability of social media and digital technologies varies between different communities, an anthropological study of the Warlpiri population illustrated how mobile phones to access social media can be seen in culturally significant ways. Warlpiri tend to use mobile phones to “intensify family intimacy” and stay connected to kin who may live far away [20]. Mobile phones are viewed as extensions of person and may be shared by family members. Cultural gender norms are also reflected in mobile phone practices; when people of the same age share a mobile phone, they tend to be of the same gender. This demonstrates how the Warlpiri population has incorporated mobile phones into their lives in ways that fit their community’s cultural practices [20].

### Use of social media for social marketing and health promotion programs

There were several reports describing use of social media for the marketing of health and the reader is referred to a recent review of peer-reviewed literature which specifically looked at the evidence for social media and mobile technologies used in health promotion, intervention, self-management and health service delivery with regard to smoking cessation, sexual health, and otitis media [26]. The No Smokes campaign utilizes multimedia and involves an interactive anti-smoking website targeting Indigenous young people. It was developed using input from Indigenous young people about what attracts them to a message and to ensure that the information was delivered in culturally accessible ways [27]. This research shaped the media focused nature of No Smokes because Indigenous young people expressed that they are more drawn to multimedia, video, social networking, animation, music and mobile phones than traditional anti-smoking marketing campaigns. These multimedia forms are more widely accessible because they minimize language and literacy barriers [13, 14, 27].

Hitnet kiosks are another example of health promotion efforts that take advantage of the multimedia nature of digital technology and social media to engage the young Indigenous population. The Heuristic Interactive Technology network (Hitnet) developed kiosks that use audio-visual touch interfaces to address issues such as smoking, sexually transmitted infections (STI), teenage pregnancy, depression and suicide through culturally based digital storytelling [28, 29]. Hitnet has placed these kiosks in some of Australia’s most remote areas and in prisons to increase the appeal and spread of health promotion efforts. The kiosks include connections to Facebook, Twitter and YouTube that can update local community content and applications that can be downloaded to personal mobile devices [29].

Online interaction can also be beneficial for Indigenous young people who struggle socially as it “allows for those with lesser social skills to tap into potentially supportive networks and develop transferrable skills for offline engagement” [1, 14, 19]. Building on opportunities to put control in the hands of the user, the Young and Well Cooperative Research Centre uses social media in its health promotion efforts. The Centre is “developing virtual mental health resources for Indigenous youth in remote communities” to connect individuals and their health care providers to online collections of data about sleep, weight, physical activity, etc. [28]. The resource is envisioned to be more of a social network that empowers the individual rather than a traditional health care intervention with an expert/client power relationship. In this way, the power and control is in the hands of the Indigenous young people, rather than the expert, which is typically a non-Indigenous person [28].

The ‘Rewrite Your Story’ campaign also focuses on regaining power and control in decision-making through the use of social media. The anti-smoking initiative focuses on sixteen local community Ambassadors who share their stories via video about how and why they gave up smoking. These videos are featured online and the campaign utilizes the idea of rewriting your story by communicating and sharing via social media channels such as Twitter and Facebook [30]. The Aboriginal Health and Medical Research Council’s ‘It’s Your Choice, Have a Voice’ campaign focused on empowering and educating Indigenous young people to make informed choices about sexual and reproductive health through active engagement, information sharing and activity on a specifically created Facebook page [31].

One research study discussed the incorporation of social media into the ArtsHealth Program, which aims to increase the proportion of Indigenous women who seek earlier and more regular antenatal care. All ArtsHealth participants in the rural settings were linked to the Facebook page and accepted ArtsHealth as a “friend.” Many participants were more responsive in communications via a Facebook inbox compared to a phone call or text message. This may be because Facebook is free to use and is easily accessible on a number of mediums such as Smartphone, tablet, laptop and personal computer. This shows that many Indigenous people engage with social media, sometimes more than they do with other research outreach methods [32].

Internet-based technologies may provide a way to bridge the gap between Indigenous and non-Indigenous students [1]. For example, a school for disadvantaged young women in Sunnybank, Queensland found “that even where English and math skills are lacking, Indigenous students have a ‘very solid grasp of technology’ despite not having a computer at home” [19]. Indigenous youth’s familiarity with mobile technologies also allows opportunities for alternative forms of learning and literacy such as digital storytelling [1, 13, 28]. A project that incorporates digital technology as part of education was the Cherbourg MoJo Project at TAFE Queensland South West, which focused on teaching mobile journalism (using iPhones to tell positive stories about their communities) to Indigenous young people with low levels of literacy and numeracy. The young people gained job skills and improved their reading, writing, and math abilities [28, 33].

Social media may also be used to foster healthy dialogue and interaction between Indigenous and non-Indigenous communities, which is considered essential for reconciliation [13, 28].

### Barriers to social media use

Although many Indigenous young people have been rapid adopters of new digital technologies including Smartphones, access can be problematic with factors such as remoteness, socioeconomic status, family structure, education level and employment status being barriers to use [19, 32]. Many Indigenous young people do not have computers or Internet access at home [16, 19]. Therefore, in some cases/places they may be less familiar with social media and digital technology than others [19].

### Negative uses of social media

Although there are many positive impacts of social media use among Indigenous young people, there are negative impacts as well, including cyber bullying, cyber racism and the exchange of sexually explicit content between minors. In many Indigenous communities, there is a generational gap in knowledge and use of social media between Indigenous young people and their parents and Elders who are often less familiar with these digital technologies [1, 19]. Kral (2014) observes that communication, increasingly mediated by technology, has disturbed traditional forms of interaction in the Western Desert community. Previously typically incorporating gesture, sign, and gaze, communication via written messages reduces the capacity for traditionally socially sanctioned forms of conflict resolution and social control by the older generation [21] This means that cyber bullying can go on unaddressed and even result in severe outcomes such as suicide if family members are not aware of young people’s activities on social media. Educational programs are needed to raise awareness of issues like cyber bullying and cyber racism in order to ensure that parents, adults, community leaders and Elders in remote locations have opportunities to learn about social media use and the potential negative effects it can have on individuals, families and communities [14, 19, 20].

Another negative effect of social media is the increased connectivity between people who live far away from each other, so that conflicts that previously remained local can spread as young people call, text or inform kin in other places about conflicts. A social networking site called Diva Chat has brought about substantial conflict among the Warlpiri population as well as in other Indigenous populations [20]. Diva Chat is a public and free to use social networking site that can be used anonymously and therefore provides a forum where some people partake in socially and culturally inappropriate behaviours because they are effectively invisible. These behaviours include illegal sex-texting, cyber bullying and posts that are meant to incite violence between feuding families. Both community and legal efforts have been made to prevent the further abuse of Diva Chat and its negative impacts. Negative experiences like this have led other communities which don’t yet have mobile connectivity to consider the potential negative effects of digital technologies before adopting them in their communities [20].

Cyber racism has been another negative consequence of social media use. Based on research with a survey group of Indigenous young people in Victoria, online racism is experienced regularly by them [1, 34]. Educational programs that focus on cyber bullying and cyber racism are one potential option to promote the safe use of social media among Indigenous young people [14, 19]. Policy is another way to tackle cyber racism, but the Australian government has done little to take down user-generated content that is in violation of the Racial Discrimination Act when it relates to overseas hosted websites like Facebook [34].

Sex-texting or ‘sexting’ is considered to be child pornography if it contains sexually explicit images of minors and is another negative use of social media common in youth across Australia [14, 20, 35]. However, sexting is hard to monitor and legal enforcement in Australia has been inconsistent. One approach addressing issues of cyber bullying, cyber racism, and sexting is an educational and community-based perspective. For example, the *Be Deadly Online* program has been launched to address cyber safety and specifically targets Indigenous young people through a culturally relevant approach [14]. The program was created in several Indigenous communities across Australia and promotes that online business is everyone’s business, cautioned users to be mindful that use creates an online digital footprint, and offers positive, practical advice on playing smart online through a series of short animations, posters and a behind-the scenes ‘making of’ video.

## Discussion

Social media and digital technology use among Indigenous young people in Australia is understudied, with research to date largely in the anthropological and social science disciplines. However, it is clear that uptake of social media has been rising rapidly, with Indigenous youth avidly utilising it in ways that suit their identity, culture and interactions with the wider world and with scholars and Indigenous activists beginning to consider the place of social media and its potential [36].

This review utilised a search based upon relevant databases. Informit and Google Scholar both have access to such large quantities of information and the data searches were sorted by relevance so the titles and abstracts of the first 50 or 100 results respectively were examined for relevance with most of the included articles appearing in the top part of the search as expected based on a search sorted by relevance. It is likely that considerably more material exists, particularly in the grey literature. Nevertheless, clear themes and uses of social media in relation to Indigenous young people.

Indigenous young people use social media to help form, affirm and strengthen identity, to feel a sense of power and control over their own lives and to make and continue community and family connections. This shows the potential for social media to be used in beneficial ways. Multimedia and the social media are culturally compatible for Indigenous young people and offer the opportunity to explore and express their identities online in ways that may not be possible in mediums such as written text [1, 17]. Social media can bring together Indigenous people who otherwise may not know each other or have been disconnected in the past, but who now have the opportunity to form a community, based on their shared Indigenous identity [1, 17]. Forming communities through social media can act as a process of uniting and healing for the Indigenous community. It is known that strong cultural identity is linked with greater participation, with achievement in education and training [37] and is a protective factor against self-harm in Indigenous young people [38]. Strengthening identity through social media may therefore offer an opportunity to help improve educational and health outcomes among Indigenous young people. There was good evidence for social media use and uptake in both remote and urban environments, suggesting that it could be utilised in settings across Australia as part of education, engagement and health promotion interventions.

Indigenous Australians have also used social media to transmit intergenerational knowledge, which may improve personal, family and community relations between the young and old generations [1, 13, 16]. Social media can enable young and old Indigenous people to reconnect and understand each other better through collaborative efforts between the generations - utilizing the skill over digital technology of the young people and the knowledge and wisdom of culture, language, Country and traditions of Elders. Younger people can express themselves and share their stories, ideas, views, photos and experiences through social media, which may help older generations to better understand where they are coming from. For example, research on Inuit youth in Canada included students making a short video about what is important to them which was shown at an important community meeting. As Garakani explains “community members, especially the elders, were touched by the videos and realized that they had underestimated the degree to which the youth were attached to the land, culture, language, and traditions” [39].

Social media also provides opportunities for the transmission of knowledge about Indigenous people to non-Indigenous people to help them better understand Indigenous cultures, perspectives and the challenges that Indigenous young people face. Improved communications and relationships between Indigenous young people and the broader community are important precedents to understanding and addressing broader social exclusion and disadvantages that Indigenous youth often experience. Thus, encouragement from community leaders, Elders, family members and others assists some school-aged students to complete Year 12 [40]. Positive experiences at school are protective against structural social issues so effective implementation of social media and digital technologies in the school environment offers the potential for improving Indigenous young peoples’ experiences at school [39].

The confidence that Indigenous young people have when approaching the use of social media invites its potential use in other arenas where they may not normally be comfortable participating. One such example is in research. A study of Inuit youth in Canada successfully used technology to gain feedback from students during the study. As Garakani states, “We observed the students’ ease in using the technology, which served as a non-threatening informal activity, allowing them to express themselves, without the barriers of language and other research formats” [39]. Increasing use of social media to document and reflect on what is important to themselves potentially gives researchers greater opportunities to work collaboratively with young people [18]. Further research that focuses on Indigenous young people could inform future programs and initiatives which use social media.

Social media is multi-dimensional in nature and related to many aspects of society, including education, policy, communication and broader community. It offers unprecedented opportunities to be exposed to learning and to interfacing with the best and worst of the wider world. Just as there are positive possibilities with social media, there are negative impacts of use such as cyber bullying, cyber racism and sexting which create concern. Social media can be used in ways which exacerbate negative images, put pressure on young people to conform and limit individuals from exploring and expressing their complex, rich and multiple identities [17]. The worldwide application of social media makes its regulation more challenging and problematic [14, 34]. While these issues are by no means limited to the Indigenous population, negative use of social media which undermines identity, control and sense of community and family connection have the potential to be particularly damaging to young people who are already vulnerable.

Therefore, programs and health promotion efforts that incorporate social media and use active engagement to promote feelings of high self-esteem, power and control and resilience have the potential to reach Indigenous young people in effective ways to increase self-efficacy. However, it is important that digital health promotion efforts do not revert back to health promotion that focuses solely on changing individual’s behaviours. Rather, digital health promotion needs to embrace the wider approach to behavioural interventions that includes social determinants of health as influential factors to individuals’ health and wellbeing [41]. A recent review examines peer-reviewed evidence for the effectiveness of social media and mobile technologies in health promotion (globally) and uses of social media to reach Indigenous Australians and mobile apps produced by Australian health bodies for health promotion efforts around smoking cessation, sexual health, and otitis media. The authors identified five Australian projects with significant social media health components targeting the Indigenous Australian population for health promotion purposes and four mobile software apps that met their criteria for inclusion but reported there was no evidence of benefit found for these projects [26].

## Conclusions

This review set out to explore the use of social media in a particular target group, namely Indigenous young people, including positive and negative impacts of social media use and how this potentially impacts health and health promotion approaches. It has shown that despite some challenges with telecommunications access and the social disadvantages experienced by many Indigenous youth, social media presents opportunities given it can have great significance in the lives of Indigenous young people. Themes that were identified in this literature review were personal and Indigenous identity, power and control, cultural compatibility of multimedia platforms and community and family connections, all indicating that social media can be used for positive benefits by Indigenous young people. Awareness and education about the negatives uses and impacts of social media are required to ensure that it is not used to exacerbate the poor social and emotional wellbeing of Indigenous young people, particularly those who experience social disadvantage and are at greater risk. Use of technology for social connection purposes does not necessarily mean that these technologies can be used for effective health interventions and there is a need to evaluate the impact of any interventions that utilise social media as a component.

The high level of uptake and involvement by Indigenous young people in digital technology and social media suggests that social media may be useful in a research setting, including for recruitment, intervention, program implementation, monitoring and evaluations. Social media could potentially help increase the involvement of Indigenous young people in research which has often been difficult to achieve and enable a more comprehensive investigation of aspects of the thinking of Indigenous youth, particularly in identifying solutions to address problems that they face in their lives. More information is needed about the associations and determinants of use and ways to maximize the positive potential of social media in the lives of Indigenous young people while limiting the negative impacts.

The high utilization of social media among young Australians, both Indigenous and non-Indigenous, creates potential opportunities to bridge the social, educational and health gaps between Indigenous and non-Indigenous young people and to enable identities, power and control and community and family connections to be shared, expanded, celebrated and strengthened. Because of this, health practitioners need to understand more about potential and appropriate ways to embrace social media to enhance health relevant interventions. We also need to be mindful that social media can also operate and divide people along social class lines [42]. Future research should focus on the potential of incorporating social media into socially focused Indigenous programs to increase participation and effective engagement and on building better relations and bridging the gaps that exist for Indigenous and non-Indigenous young people.
